# From SNPs to Genes: Disease Association at the Gene Level

**DOI:** 10.1371/journal.pone.0020133

**Published:** 2011-06-30

**Authors:** Benjamin Lehne, Cathryn M. Lewis, Thomas Schlitt

**Affiliations:** 1 Department of Medical and Molecular Genetics, King's College London, London, United Kingdom; 2 Social, Genetic and Developmental Psychiatry Centre, Institute of Psychiatry, King's College London, London, United Kingdom; Memorial Sloan Kettering Cancer Center, United States of America

## Abstract

Interpreting Genome-Wide Association Studies (GWAS) at a gene level is an important step towards understanding the molecular processes that lead to disease. In order to incorporate prior biological knowledge such as pathways and protein interactions in the analysis of GWAS data it is necessary to derive one measure of association for each gene. We compare three different methods to obtain gene-wide test statistics from Single Nucleotide Polymorphism (SNP) based association data: choosing the test statistic from the most significant SNP; the mean test statistics of all SNPs; and the mean of the top quartile of all test statistics. We demonstrate that the gene-wide test statistics can be controlled for the number of SNPs within each gene and show that all three methods perform considerably better than expected by chance at identifying genes with confirmed associations. By applying each method to GWAS data for Crohn's Disease and Type 1 Diabetes we identified new potential disease genes.

## Introduction

Genome-Wide Association Studies (GWAS) link genetic variants to phenotypes. One common study design in human disease genetics is to compare a group of diseased individuals (cases) to a group of healthy individuals (controls) for a large number of Single Nucleotide Polymorphisms (SNPs). The frequency of each allele is compared between cases and controls using a χ^2^ statistic, which can be transformed into a measure for the probability of the data arising under no association between disease and SNP (p-value). Currently, GWAS are carried out using microarray technology, genotyping up to one million SNPs in parallel. Because a statistical test is performed for each SNP, careful multiple hypothesis testing procedures are employed to ensure the identification of association signals with genome-wide significance, typically with a p-value p<5•10^−8^ [1]. In most GWAS only a few SNPs pass this correction and although this approach has led to the discovery of several novel disease-linked variants, it ignores thousands of SNPs with “suggestive” p-values that fail to reach the stringent threshold for genome-wide significance, but may reflect evidence for association. Several approaches try to make use of these “suggestive” p-values through the incorporation of prior biological knowledge [2], [3], [4], [5], [6], [7], [8], [9], [10], [11], [12]. The best known is Gene Set Enrichment Analysis (GSEA) [3], [13], which assesses whether predefined sets of genes are overrepresented within a sample. Genes that are members of the same gene-set are typically involved in a common biological process as defined by e.g. the Gene Ontology [14] or biological pathways as defined by databases such as KEGG [15]. In a similar way, protein networks have been consulted [10], [11] with the objective of identifying subnetworks of interacting proteins. Individually none of the proteins within such a subnetwork might be significantly associated, but overall a subnetwork might show statistically significant association with a disease.

All of these studies face very similar methodological problems: GWAS report association for individual SNPs, whereas functional information typically exists for proteins or genes. Therefore SNPs have to be assigned to genes and their individual association signals combined. This can be done in different ways and one must take into consideration that the number of SNPs per gene can vary to a great extent. The most widely used approach is to take the most significant p-value per gene [2], [3], [4], [5], [6], [7], [8]; however this can introduce a substantial bias in the downstream analysis if the number of SNPs per gene is not controlled for [9]. In this work we systematically compare three methods to analyse GWAS data at the gene level. We also propose a way to control for differences in the number of SNPs per gene based on permutations of the disease status and demonstrate its effectiveness. Based on GWAS data for Crohn's disease (CD) and Type 1 Diabetes (T1D) genotyped by the Wellcome Trust Case Control Consortium [16], we evaluate the performance of the different methods using sets of disease genes that were identified and replicated by the most recent meta-analyses [17], [18].

## Methods

### Quality Control and Association Testing

GWAS of seven diseases have been performed by the WTCCC [16]. Approximately 3,000 shared controls and 2,000 cases were genotyped for seven diseases, including Crohn's Disease (CD) and Type 1 Diabetes (T1D), on the Affymetrix GeneChip 500K Mapping Array Set. We re-analyzed the WTCCC I data using PLINK v1.06 [19]. In addition to SNPs and individuals in the exclusion lists provided with the genotyping data, we applied more stringent quality control criteria than the original study, because our analysis includes moderate associations which are more susceptible to study biases. Based on the pooled case/control dataset we excluded SNPs with Hardy-Weinberg equilibrium p<0.001, a minor allele frequency of less than 0.01 or genotyping call-rates of less than 0.97. Association testing was performed using the Cochran Armitage trend test (1df). We manually checked the most strongly associated SNPs for every disease to ensure consistency with the original WTCCC I results. To take into account inflated test statistics caused by population stratification we corrected test statistics using the genomic control metric λ_median_ [20]. The estimated λ_median_ (for simplicity denominated as λ) for CD (λ = 1.12) and T1D (λ = 1.06) are in good agreement with the original values reported by the WTCCC (λ = 1.11 and λ = 1.05 for CD and T1D, respectively). For both diseases, 500,000 permutations of the disease status were performed using the PLINK max(T) permutation method and association p-values were calculated. Table 1 summarises the GWAS data analysis for CD and T1D.

**Table 1:** Overview statistics of the analysed GWAS datasets and the gene to SNP assignment for Crohn's Disease (CD) and Type 1 Diabetes (T1D).

	CD	T1D
Number of cases before QC	2,009	2,000
Number of cases after QC	1,752	1,964
Number of controls before QC	3,004	3,004
Number of controls after QC	2,938	2,938
Genomic Control metric **λ**	1.12	1.06
Protein-coding genes on chromosome 1–22	20,919	20,919
Protein-coding genes after SNP to gene assignment	17,006	17,006
Protein-coding genes after QC	16,326	16,146
SNPs on the Affymetrix GeneChip 500K Mapping Array Set	500,568	500,568
SNPs assigned to genes (chromosome 1–22)	290,571	289,098
SNPs assigned to genes after QC	227,418	225,973

To further assess the effect of population stratification on our analyses we performed principal component analysis (PCA) of the CD and T1D data using EIGENSTRAT [21]. We then performed association testing using logistic regression to incorporate the first two principal components as covariates. For both diseases, 1,000 permutations of the disease status were performed using logistic regression and the PLINK max(T) permutation method.

### Gene to SNP assignment

A tab-delimited text-file (seq_gene.md) containing genomic coordinates for all genes was downloaded from the NCBI ftp-server [22] in November 2009. Only entries for the human reference sequence (NCBI assembly GRCh37) and protein-coding genes were retained. Genes mapping to sex-chromosomes, the mitochondrial chromosome, unassembled contigs or alternative haplotypes were discarded. SNPs on the GeneChip 500K Mapping Array Set were assigned to the remaining genes. Because this genotyping platform is based on the previous assembly of the human genome (NCBI 36) all SNP positions were converted to the latest assembly using the “Lift-Over” tool on the GALAXY website [23]. SNPs were assigned to a gene if they are located within its primary transcript or 40 kilobases (kb) upstream or downstream. These boundaries are chosen based on the distribution of association signal with respect to protein-coding genes [24]. When a SNP could be assigned to multiple genes because of overlapping flanking windows, the closest gene was chosen.

The WTCCC study found the strongest association signal for Type 1 Diabetes (T1D) within the Major Histocompatibility Complex (MHC) region on chromosome 6. The MHC region has high levels of linkage disequilibrium (LD) and harbours many genes. This causes the association signal to be spread over many genes, thereby artificially inflating the number of genes with associated SNPs. We therefore excluded the MHC region (chromosome 6, position 25,930,839 to position 33,495,825, NCBI assembly GRCh37) in all analyses of the T1D dataset, which removed 1,473 SNPs and 185 genes. In total, approximately 290,000 SNPs were assigned to 17,000 protein coding genes. Table 1 summarises the SNP to gene assignment for CD and T1D.

### Assessment of LD on SNP to gene assignment

In order to assess the effect of LD we repeat our analyses, but take into account LD to extend the assignment of SNPs to genes. We use PLINK v1.06 [19] to obtain a list of SNP pairs in LD (r^2^>0.8) based on the GWAS data for CD and T1D [16]. SNPs are added to the initial assignment if they are in LD (r^2^>0.8) with a SNP in a gene or its 40 kb flanking windows, including SNPs that have already been assigned to other genes. Taking into account LD adds approximately 6,000 (2%) additional SNPs to the analyses.

### Deriving a gene-wide test statistic for each gene

Each gene has *n* SNPs assigned to it with *n ∈* ***N_0_***. Let the test statistics in the gene be *T_i_, i* = 1, … *n*. Under the null hypothesis of no association, *T_i_* has a χ_1_^2^ distribution (χ^2^ distribution with one degree of freedom); high values of *T_i_* indicate evidence for association. To obtain a gene-wide test statistic, we use three summary statistics for *T_i_*:

1. **maxT:** the maximum value of *T_i_* (maximum χ_1_^2^ value) for each gene is chosen;
2. **meanT:** the arithmetic mean test statistic (mean χ_1_^2^ value) for each gene is calculated;
3. **topQ:** the highest quartile of all test statistics *T_i_* (highest quartile of all χ_1_^2^ values) in a gene are selected and their mean is calculated. If *n* is not a multiple of 4 the number of SNPs considered for topQ is rounded up to the next integer (e.g. if a gene has 5 SNPs the mean of the largest two test statistics is calculated).

### Deriving an empirical p-value (*p_emp_*) for each gene

We derive test statistics for each gene in the observed dataset and in 500,000 randomised datasets derived from permutations of the disease status. For each gene we tabulate the number of permuted data sets in which we observe a higher gene-wide test statistic than in the observed data set, thus deriving an empirical p-value *p_emp_*.

Because we compare observed and permuted test statistics for every gene, a significantly associated gene requires a *p_emp_* value that is also controlled for the number of genes tested. Assuming there are approximately 20,000 protein-coding genes in the human genome, a Bonferroni correction requires a p-value threshold of p_emp_  = 0.05×1/20,000  = 2.5×10^−6^. In order to be able to obtain p-values of that magnitude we perform 500,000 permutations of the disease status. Empirical p-values are derived for each gene for all three methods to derive gene-wide test statistics.

### Uncontrolled vs. empirical p-value

To compare the different methods we rank genes for each gene-wide test statistic method. This is done before and after deriving *p_emp_* values (i.e. controlling for the number of variants per gene and LD) resulting in six different sets of ranks. When *p_emp_* values are identical for two or more genes we use the gene-wide test statistics to resolve ties. Based on the ranks we calculate pairwise Spearman rank correlation coefficients between all six sets for the top 500 genes: For each gene, we sum the ranks across all six gene sets, and select the 500 genes with the highest summed ranks.

To analyse the effect of deriving *p_emp_* values for individual genes we convert the gene-wide test statistics to p-values assuming test statistics have a χ_1_^2^ distribution. For each gene the uncontrolled p-value is plotted against the *p_emp_* value for all three methods.

### Performance

To assess the performance of the three methods for deriving *p_emp_* values we calculate Receiver Operating Characteristic (ROC) curves, which estimate the accuracy of a prediction by comparing the True Positive Rate (TPR  =  True Positives/Positives) with the False Positive Rate (FPR  =  False Positives/Negatives) [25]. In this analysis we used as positives a list of successfully replicated disease genes from meta-analyses of T1D [18] and CD [17]. We only chose loci that either contain a single gene or a for which a unique candidate gene has been proposed [17], [18]. This results in 39 and 27 true positive genes for CD and T1D, respectively (Tables 2, S1 and S2). We assume that all other genes are negatives. We rank all genes within both lists (positives and negatives) by their *p_emp_* values, and used their gene-wide test statistics to resolve ties when *p_emp_* values are identical for two or more genes. For each gene the relative rank within the positives is plotted against the relative rank within the negatives to derive the ROC curve, and the areas under the curve (AUC) were calculated.

**Table 2:** Replicated Disease Genes for Crohn's Disease from [17] and their ranks for each method.

		Rank of gene for		
hgnc	Number of SNPs per gene n	rank maxT	rank meanT	rank topQ
***NOD2***	13	1	3	2
***ATG16L1***	11	2	1	1
***IL23R***	21	3	4	3
***NKX2-3***	26	5	5	5
***PTPN2***	20	7	11	10
***IRGM***	5	8	2	6
***ZNF365***	91	18	149	67
***GCKR***	6	31	81	76
***CREM***	12	34	59	46
***C13orf31***	6	43	136	78
***IL12B***	14	55	45	94
***SP140***	18	64	110	56
***CDKAL1***	127	83	486	248
***C11orf30***	22	336	164	180
***VAMP3***	1	357	356	348
***CCR6***	14	602	291	319
***DNMT3A***	9	612	667	399
***MTMR3***	23	827	788	715
***FADS1***	3	980	921	1002
***NDFIP1***	19	1020	754	506
***TAGAP***	4	1169	3445	1203
***IKZF3***	8	1192	4281	995
***DENND1B***	22	1337	3818	1855
***THADA***	59	1500	1157	1206
***JAK2***	17	2074	3065	3038
***PTGER4***	4	2620	4084	2622
***PTPN22***	4	3457	2465	3498
***SMAD3***	42	4071	11565	9918
***CPEB4***	21	4108	4080	3842
***ICOSLG***	5	6041	8465	6570
***PRDM1***	18	6151	4859	5241
***IL2RA***	20	6698	8568	5229
***BACH2***	49	8022	3687	2962
***MAP3K7IP1***	6	8040	6149	6685
***PLCL1***	56	8317	3347	3754
***ICAM3***	2	9345	9596	9415
***UBE2D1***	4	10217	12176	10212
***TNFSF11***	21	11858	9547	9927
***ZMIZ1***	72	14061	5704	9596

All scripts written for the analyses presented are available from authors upon request.

## Results

### Number of SNPs per gene

**Figure 1:**
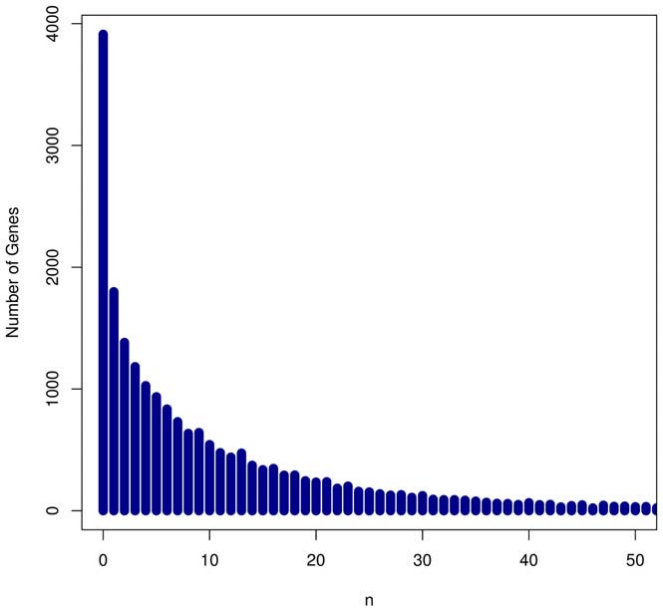
Distribution of the number of SNPs assigned to genes. We assigned SNPs on the Affymetrix 500K genotyping array to protein-coding genes. SNPs were assigned to a gene if they are located within the transcribed region or within a 40 kilobase flanking window around the transcribed region. Where flanking windows overlapped SNPs were assigned to their closest gene only.

The Affymetrix 500K GeneChip includes approximately 500,000 SNPs distributed over the whole genome. We assign these SNPs to their closest protein-coding gene if a SNP is located less than 40 kb from a gene. Approximately 290,000 SNPs were assigned to genes, of which 227,000 were left after QC for specific disease data sets (Table 1). Genes vary substantially in size, which leads to different numbers of SNPs assigned to each gene (Figure 1). Of 20,919 protein-coding genes 17,006 have at least one SNP assigned; most of these genes (∼77% or 13,083 genes) have fewer than 10 SNPs; 6.5% (1,097 genes) have more than 50 SNPs. The largest number of SNPs assigned to a single gene is 1,008 (*CSMD1*, gene length: 818 kb).

We performed analyses of GWAS data for both Crohn's Disease (CD) and Type 1 Diabetes (T1D). In the following section we present results for CD. Results for T1D are comparable and presented in supplementary material.

### Deriving a gene-wide test statistic for each gene

**Figure 2:**
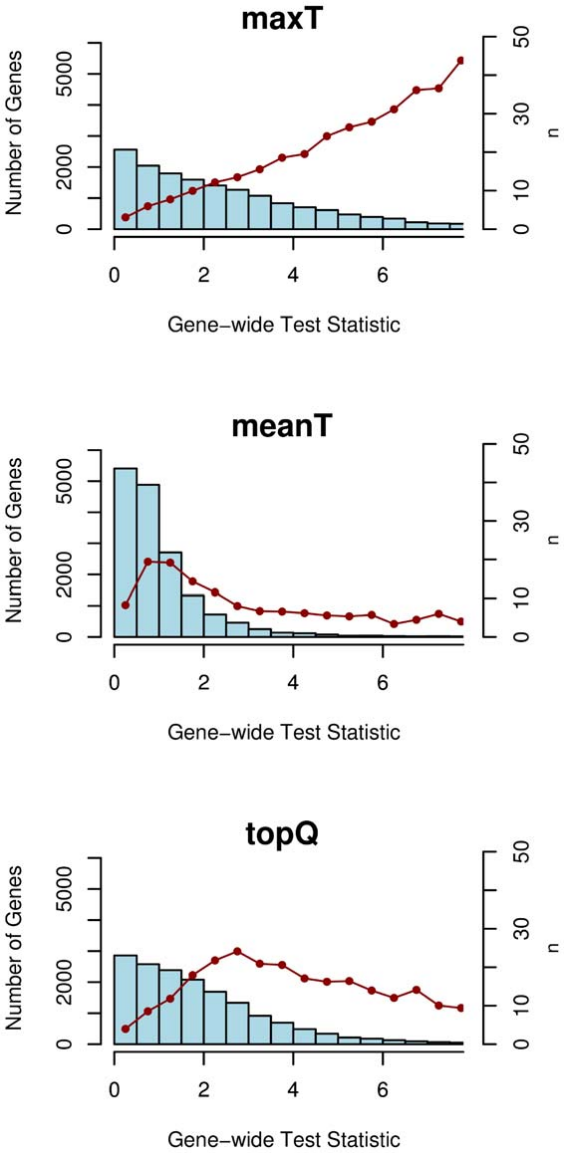
Confounding effect of the number of SNPs per gene (Crohn's Disease). Multiple test statistics are combined for each gene using three different methods (maxT, meanT, topQ). For each method, the gene-wide test statistic is correlated with the number of SNPs per gene. For these histograms, genes are binned according to their gene-wide test statistic (left axis). The red dots show the mean number of SNPs per gene for every bin (right axis).

To measure association of a SNP with the disease we compare genotype frequencies between cases and controls and calculate a genomic control-corrected test statistic based on an Armitage trend test for every SNP. To obtain a gene-wide measure of association we first derive three summary statistics: **maxT** (the maximum test statistic for each gene), **meanT** (the mean test statistic for each gene), and **topQ** (the mean of the highest quartile of all test statistics in a gene). Here we illustrate how each summary statistic is subject to confounding factors that have to be controlled for. The gene-wide test statistic is correlated with the number of SNPs per gene, *n* (Figures 2 and S1), as follows.

- For **maxT** the test statistic increases approximately linearly with *n* (Pearson correlation coefficient r = 0.36). Even if there is no association, genes with many SNPs assigned are more likely to have a SNP with a high test statistic, by chance.
- A different effect occurs for **meanT**, whereby genes with many SNPs tend to have gene-wide test statistics close to one, whereas genes with few SNPs tend to be at the extremes of the distribution, i.e. to have either very low or very high gene-wide test statistics. Under the null hypothesis of no association, the test statistic has a χ_1_^2^ distribution, with a mean of 1. When calculating meanT, genes with more SNPs are therefore likely to have gene-wide test statistics close to 1, whereas genes with few SNPs are more affected by individual SNPs with extreme test statistic.
- An effect similar to meanT is observed for **topQ**: Genes with fewer SNPs tend to have extreme gene-wide test statistics whereas genes with many SNPs tend to have a gene-wide test statistic close to χ^2^≈3. This value is higher than for the meanT method since only the top 25% of SNPs per gene are selected.

### Deriving an empirical p-value for each gene

**Figure 3:**
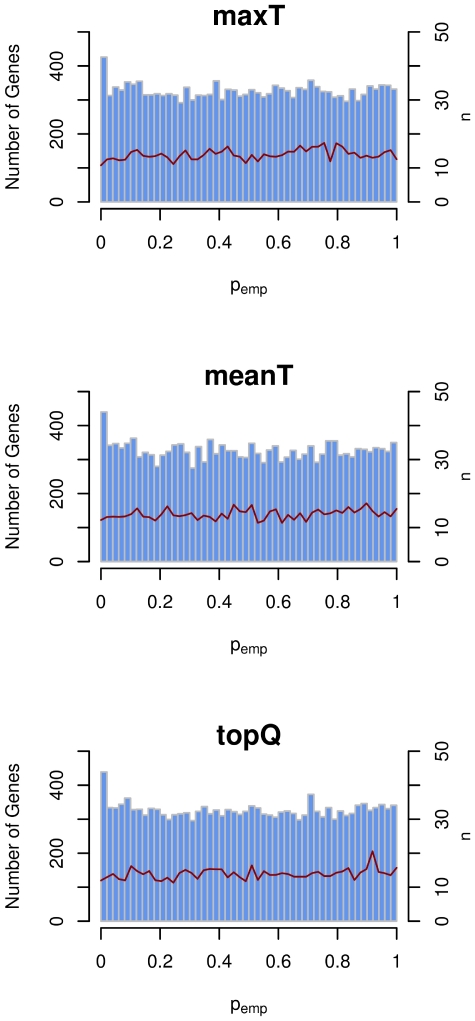
Distribution of empirical p-value (*p_emp_*) for Crohn's Disease from 500,000 permutations of the disease labels. Genes were assigned to 50 bins according to their p_emp_. Histogram shows the number of genes with *p_emp_* values (left axis). The red line shows the mean number of SNPs per gene for every bin (right axis). In contrast to the gene-wide test statistics we observe no correlation of the number of SNPs per gene with *p_emp_* for any method. We observe an increase of genes with very low *p_emp_* values caused by the actual association signal.

The distribution of the summary statistics for each gene is not known and impossible to derive analytically, since it depends on the pattern of LD within each gene. We therefore derive an empirical p-value *p_emp_* for each gene from permuted datasets (see Methods). By comparing the observed to the permuted test statistics we maintain LD structure and account for differences in the number of SNPs per gene. The observed *p_emp_* values are appropriately controlled for the number of SNPs per gene; we observe no correlation between the number of SNPs per gene and the *p_emp_* value (Figures 3 and S2). For each of the three methods to combine test statistics, the *p_emp_* values are approximately uniformly distributed. The high proportions of very low *p_emp_* values (Figures 3 and S2) are likely due to true association signal.

### Uncontrolled vs. empirical p-value

Although different methods yield different levels of association for a given gene, the results are correlated. Between the three methods to derive p_emp_ values, we observe an average Spearman rank correlation coefficient of 0.74 when considering the top 500 genes (Tables S1 and S2). The average Spearman rank correlation coefficient between the three methods before deriving p_emp_ values (i.e. controlling for the number of variants per gene and LD) is only 0.30, which reflects the different biases introduced by the methods to derive gene-wide test statistics

**Figure 4:**
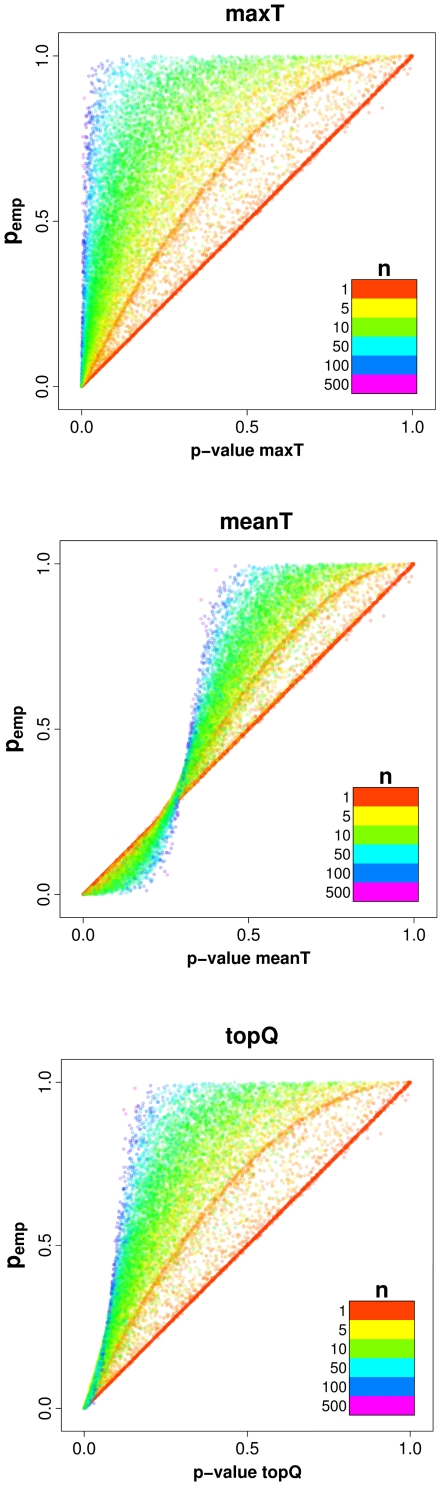
Empirical p-values vs. uncontrolled p-values (Crohn's Disease). For each gene the *p_emp_* is plotted against the uncontrolled p-value (based on the gene-wide test statistic). Each point represents a gene and is coloured according to the number of SNPs assigned to a gene (*n*). Genes with few SNPs have *p_emp_* values similar to the uncontrolled p-value and therefore cluster along the diagonal. For genes with higher number of SNPs the distribution depends on the method to combine test statistics.

The *p_emp_* values are controlled for the number of SNPs per gene and the correlation structure, but how does the control affect individual genes? To address this question, we convert the combined test statistics to p-values assuming test statistics have a χ_1_^2^ distribution. These uncontrolled p-values are plotted against the *p_emp_* values for all three methods (Figures 4 and S3):

- For the **maxT** method, genes with many SNPs (large *n*) are more likely to have a high test statistic and therefore a low uncontrolled p-value. When deriving *p_emp_* values we control for *n*. The control has very little impact on genes with *n* = 1 and in that case the empirical and the uncontrolled p-values are very similar (lying along the diagonal in Figures 4 and S3). For genes with higher *n* the control is stronger and *p_emp_* values are higher than the uncontrolled p-values.
- For **meanT** we observe a sigmoid-like distribution. That is explained by the effect of varying *n*: We compare permuted to observed test statistics. If there is no association the expected test statistic is 1. Therefore the expected meanT values for the permuted datasets are 1, i.e. with increasing *n* the permuted meanT is more likely to be 1. For genes with large *n* this leads to extreme *p_emp_* values when we compare observed to the permuted meanT. As a result the distribution for genes with large *n* shows a stronger curvature than for genes with small *n*. When the observed meanT value is 1 (uncontrolled p-value  = 0.317) the control is (on average) not affected by *n*. Therefore the points representing genes with different *n* overlap at meanT = 1.
- The distribution for **topQ** is similar to maxT, but the gradient for genes with many SNPs is less steep.

### Performance

**Figure 5:**
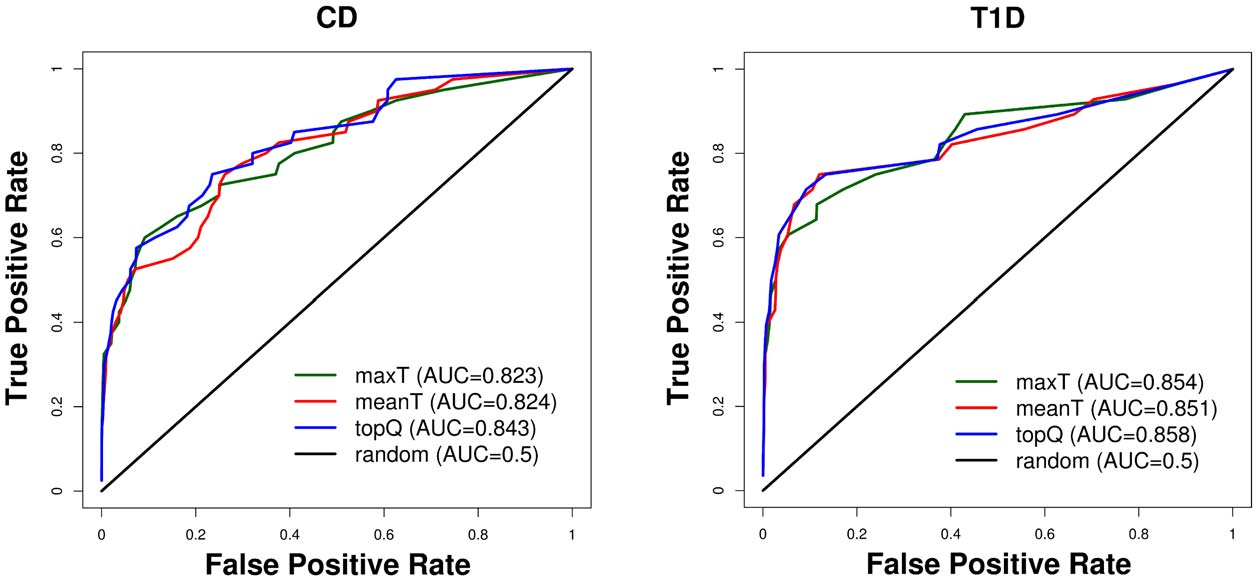
Receiver Operating Characteristic (ROC) curves for Crohn's Disease (CD) and Type 1 Diabetes (T1D). To assess the performance of different methods to combine test statistics we plot the proportion of confirmed disease genes (True Positive Rate) against their rank within the whole set of genes (False Positive Rate).

To assess the performance of the different methods of combining test statistics we plot Receiver Operating Characteristic (ROC) curves for CD and T1D (Figure 5) using two sets of confirmed disease genes [17], [18] under the assumption that all other genes are not associated (see Methods). The known disease genes are based on meta-analyses CD [17] and T1D [18]. Based on genomic loci that successfully replicated the authors selected the most likely candidate gene considering known involvement in the immune system, association with other auto-immune disorders and location of the most strongly associated SNP. Although the resulting gene list may contain genes which are not associated with the trait, it is the best currently available dataset to assess the performance of our methods for measuring genetic association at the gene-level.

All three *p_emp_* methods give considerably better results than expected by chance. For both diseases the topQ method performs slightly better than maxT and meanT, although all three methods perform similarly with differences in the areas under the curve (AUC) of less than 2%. The performance of the different methods for the two diseases might depend on the number of SNPs assigned to the known disease genes. For genes with many SNPs the association signal can get diluted, as it is the case for the CD disease gene *ZNF365*, which has 91 SNPs (Table 2). Its maxT is 23.74 which corresponds to *p_emp_* = 0.0001, but the meanT and the topQ for this gene are 2.46 (*p_emp_*  = 0.0041) and 8.32 (*p_emp_* = 0.0010), respectively. Consequently the performances measured here by the AUCs depend on the properties of the known disease genes and we can only assume that they are characteristic for disease genes that have not been identified yet.

Several known disease genes were consistently ranked very low by all three methods (Table 2). For some of these genes the associated SNPs are over 40 kb from the gene (e.g. *PTPN22*), or the associated SNP is located in the adjacent gene (e.g. *ORMDL3).* Other confirmed disease genes were ranked low because the associated SNP has not been genotyped by the WTCCC (e.g. *JAK2)* or did not show any association (e.g. *PLCL1*).

### Linkage Disequilibrium

Our analysis is influenced by linkage disequilibrium (LD) and some of the top ranked genes (Table 3) are part of the same LD region, reflecting the fact that a true association signal could extend over a large region of the genome if it falls into a large LD block. Most of the SNPs in such a region would appear to be associated with the phenotype which can result in several genes with significant empirical p-values. For example, *CYLD* and *SNX20* have *p_emp_* values smaller than 5.4×10^−5^; they are located upstream and downstream of *NOD2* and are located in the same LD block as *NOD2*. Their association is most probably an artefact of the confirmed association of the *NOD2* gene [26], [27], [28]. To further assess the impact of LD on our analyses we extended the initial gene to SNP assignment. In addition to SNPs located within the gene or a 40 kb flanking window we include SNPs in LD (r^2^>0.8) with any SNP in this region. This increases the average number of SNPs per gene to 15.5 (from 13.9) and the total number of SNPs assigned to genes to over 296,000 (from 290,000) (Figure S4). Including LD in the gene to SNP assignment has only a moderate effect: Although AUC values show a small increase for each method (<1.3%), only a small minority of genes is affected (Figure S5). Gene ranks obtained with and without taking into account LD are highly correlated (Spearman rank correlation r = 0.98 for each method and disease). Only 3 genes out of the top 100 have a rank above 100 when including LD (maxT for CD) and all genes discussed here and shown in the tables only marginally change their rank or p-value.

**Table 3:** The top 30 ranked genes for Crohn's Disease (CD) using the maxT method.

HGNC symbol	Chr location	Region (Mb)	n	p-value maxT	p-value meanT	p-value topQ	rank maxT	rank meanT	rank topQ
C1orf141	1p31	67.56-67.59	26	2.0E-06	8.7E-04	6.0E-06	9	60	9
***IL23R***	1p31	67.63-67.73	21	>2.0E-06	>2.0E-06	>2.0E-06	3	4	3
IL12RB2	1p31	67.77-67.86	17	6.0E-06	1.4E-03	6.8E-04	10	76	54
***ATG16L1***	2q37	234.16-234.20	11	>2.0E-06	>2.0E-06	>2.0E-06	2	1	1
USP4	3p21	49.31-49.38	5	2.1E-04	1.0E-04	1.1E-04	28	22	23
TCTA	3p21	49.45-49.45	2	1.1E-04	2.0E-06	1.1E-04	21	8	22
AMT	3p21	49.45-49.46	3	7.1E-05	2.0E-06	7.1E-05	19	7	17
DAG1	3p21	49.51-49.57	4	1.2E-04	2.4E-05	1.2E-04	23	14	24
BSN	3p21	49.59-49.71	13	1.6E-05	2.9E-04	1.6E-05	13	32	13
APEH	3p21	49.71-49.72	1	7.3E-05	7.3E-05	7.3E-05	20	19	18
IP6K1	3p21	49.76-49.82	1	2.2E-04	2.2E-04	2.2E-04	29	27	31
SLC22A5	5q31	131.71-131.73	9	1.4E-05	3.3E-04	1.4E-05	12	35	12
C5orf56	5q31	131.75-131.80	13	2.0E-05	4.0E-06	6.0E-06	15	10	8
***IRGM***	5q33	150.23-150.23	5	>2.0E-06	>2.0E-06	>2.0E-06	8	2	6
ZNF300	5q33	150.27-150.28	10	>2.0E-06	2.0E-06	>2.0E-06	6	9	7
TRIM10	6p21	30.12-30.13	2	1.9E-04	6.6E-04	1.9E-04	25	51	26
HLA-DQB1	6p21	32.63-32.63	11	2.8E-05	3.8E-04	2.1E-04	16	39	29
HLA-DQA2	6p21	32.71-32.72	29	1.1E-04	1.2E-05	1.6E-05	22	12	15
C7orf33	7q36	148.29-148.31	13	2.4E-04	4.6E-04	1.0E-04	30	42	21
LOC100130652	10p15	3.87-3.87	24	1.4E-04	8.8E-02	4.1E-02	24	1,600	809
***ZNF365***	10q21	64.13-64.43	91	5.8E-05	4.1E-03	9.6E-04	18	149	67
***NKX2-3***	10q24	101.29-101.30	26	>2.0E-06	>2.0E-06	>2.0E-06	5	5	5
SNX20	16q12	50.70-50.72	3	1.2E-05	5.4E-05	1.2E-05	11	17	11
***NOD2***	16q12	50.73-50.77	13	>2.0E-06	>2.0E-06	>2.0E-06	1	3	2
CYLD	16q12	50.78-50.84	16	>2.0E-06	>2.0E-06	>2.0E-06	4	6	4
STAT3	17q21	40.47-40.54	13	2.1E-04	2.0E-04	8.5E-05	27	26	19
***PTPN2***	18p11	12.79-12.88	20	>2.0E-06	7.9E-06	6.0E-06	7	11	10
SBNO2	19p13	1.11-1.17	3	2.0E-04	1.9E-04	2.0E-04	26	24	27
RSHL1	19q13	46.30-46.32	1	1.6E-05	1.6E-05	1.6E-05	14	13	14
ZGPAT	20q13	62.34-62.37	1	3.4E-05	3.4E-05	3.4E-05	17	15	16

Genes are ordered by chromosome and genomic position; n denominates the number of SNPs per gene. The last three columns show the corresponding ranks for the three methods. *italics*: genes that are within the true positive list.

### Population Stratification

Our primary analysis method is testing for association with the Cochran Armitage Trend Test, with genomic control correction for population ancestry, as this makes performing large numbers of permutations computationally tractable. To assess the effect of population stratification on our analysis in more detail we performed Principal Component Analysis [21] for both datasets. We repeated association testing using logistic regression and adjusting for the first two principal components (PC-correction). This reduced the genomic control measure for CD from λ = 1.12 to λ = 1.08, with no reduction observed for T1D (λ = 1.06). Adjusting for up to 10 PCs did not reduce λ any further. The correlation between gene ranks of our primary analysis and after correction for population stratification was high (CD-maxT R = 0.932, CD-meanT R = 0.942, CD-topQ R = 0.940, T1D-maxT R = 0.997, T1D-meanT R = 0.998, T1D-topQ R = 0.998). Gene ranks for CD are more affected than for T1D: out of the top 100 genes of our primary analysis, 78 are within the top 100 genes after PC-correction, and all 100 are within the top 204 genes (maxT, Figure S6). For T1D, 86 out of the top 100 genes of our primary analysis are within the top 100 after PC-correction and all 100 are within the top 143 genes (maxT, Figure S6). Correcting for two principal components only marginally affects the performance of our methods: AUC values increased by <0.6% for both CD and T1D.

### Associated Genes

All genes discussed here only marginally change their rank or p-value after correcting for two principal components or when considering LD for the SNP to gene assignment. For CD we find 7 out of 39 known disease genes (true positives) within the top 30 genes when we rank all genes based on *p_emp_* values (derived from maxT). We use their gene-wide test statistics to resolve ties when *p_emp_* values are identical for two or more genes (Table 3). The genes *STAT3* (maxT rank 27) and *SBNO2* (maxT rank 26) are located within known disease loci, but are not part of the true positive list because the association signal extends over several genes [17]. Both loci did not reach genome-wide significance in the original WTCCC study and their association was only confirmed in a more recent large-scale meta-analyses. *STAT3* and *SBNO2* can be linked to the *IL10/STAT3* anti-inflammatory pathway [29], which has been implicated with CD [2], [17], [30].

Another promising candidate for CD might be *DAG1* (dystroglycan 1), ranked 23rd for maxT. It is located within a large LD block whose association has been replicated and that encompasses about 35 genes [17]. *DAG1* is a cell surface receptor which is used by several known pathogens [31], [32] and there has been speculation about a role for *DAG1* in the uptake of *Mycobacterium avium* ssp. *paratuberculosis* and the aetiology of Crohn's Disease [33].

For T1D five out of 27 known disease genes are within the top 30 (based on maxT, Tables S3 and S4). Of the top 30 genes, 14 fall into a large LD region on chromosome 12 (position 111,348,628 to position 112,947,717), which contains 15 genes. According to Todd *et al.* [34] the most probable causal gene for this region is *SH2B3.* The authors detected a highly associated non-synonymous SNP in exon 3 of *SH2B3,* which had not been genotyped in the WTCCC study [16]. Two SNPs that were genotyped in the WTCCC are assigned to *SH2B3* and show moderate association (p = 3×10^−5^ and p = 7×10^−4^). Since 40 other SNPs in the region show stronger association, *SH2B3* is only ranked 26 (by maxT).

## Discussion

Based on GWAS data for two common diseases we present three different methods to combine individual test statistics at a gene level. For all methods the gene-wide test statistic is correlated with the number of SNPs per gene. Based on permutations of the disease status we derive an empirical p-value for each gene and show that it is controlled for the number of SNPs within the gene. To assess the performances of the *p_emp_* methods we derive ROC curves based on two sets of disease genes that were replicated in the most recent meta-analyses [17], [18]. The p_emp_ methods distinguish different genetic architectures underlying a disease: for maxT a single mutation within a gene contributes to the disease (i.e. one SNP within a gene shows association); for meanT mutations spread all over the gene contribute to the disease (i.e. all or many SNPs within a gene show association): in the case of topQ only a few mutations within a gene contribute to the disease (i.e. a subset of the SNPs within a gene show association). All three methods performed substantially better than expected by chance at identifying these genes, thus justifying our approach. The performances of the three methods were similar, demonstrating the robustness of the permutation approach. This is also reflected by the correlations between empirical p-values for each method for the top 500 genes. For some genes however, results can vary across the methods, as illustrated by *ZNF365* (Table 2). To identify all potentially associated genes, results from all methods should be considered. As the methods are correlated, integration results in a moderate increase in the number of genes. For example, the union of the top 500 genes for all three methods consists of 678 genes.

In this work we perform gene-wide analyses on two independent GWAS datasets. We observe the same overall properties for gene-wide test statistics and p_emp_-values. Furthermore for both datasets our methods successfully reproduced known disease associations showing the robustness of our approach. In addition to the methods presented here other methods have been proposed, including multi-marker association tests [35], [36], [37], [38] and variations [39], [40], [41] of Fisher's method to combine p-values [42]. Recently, two studies proposed approaches to control for confounding factors (e.g. number of SNPs per gene) which do not require genotyping data [12], [43]. Further studies will be required to determine how these methods compare.

An open problem that still has to be addressed is the effect of LD. Correlation between the SNPs of a gene can impact the combined test statistic for meanT and topQ method. Because multiple associations can be caused by a single causal SNP a high meanT or topQ might not reflect several independent associations. Correlation between the SNPs of a gene can therefore change the nature of the method to combine test statistics. Furthermore LD makes it difficult to allocate association signal to the correct gene. A number of groups have proposed computational approaches to prioritize genes within LD blocks [6], [44], [45]. They have been shown to give reasonably good results and could be combined with our approach.

Another approach is to use imputed genotypes, which will increase the density of SNPs and therefore the proportion of genes that are captured. Hong et al. [9] were able to include over 800 additional genes (5%) in their gene-wide analysis of GWAS data, but levels of statistical significance for most other genes remain unchanged compared to using genotyped SNPs only. Assigning SNPs to genes is not straight forward as regulatory elements such as enhancers can be many kilobases away from the transcribed region. In addition some disease-associated variants are located in so-called gene deserts that cannot be linked to protein-coding genes or any other functional elements. Ultimately functional studies are necessary to determine which gene is implicated in a disease process. The methodology demonstrated here is instrumental in automatically identifying the relevant genes that might be implicated in inherited disorders and provides an unbiased ranked list of genes for experimental validation.

Currently GWAS are moving from microarray based technology towards next-generation sequencing (NGS). NGS, in principle, allows for the identification of all genetic variants. As the number of genetic variants in a given individual is far higher [46] than the number of SNPs genotyped using microarray technology, the number of tests is going to increase dramatically. There is a need for new analytical methods that combine association signals over several genetic variants or all variants within a gene, particularly for rare variants which may individually lack power to show significant association. Testing for combined association of all rare variants within a gene overcomes this problem, as demonstrated for simulated data and sequence data of previously known disease genes [47], [48], [49].

With the emergence of next-generation sequencing, GWAS will increasingly be analysed on gene level. Gene-level association measurements allow the application of gene-set enrichment analysis and related methods, which will ultimately improve the understanding of the underlying molecular mechanism. The methods proposed here provide an accurate and powerful approach to summarise evidence for association within genes and could be used to design functional follow-up studies.

## Supporting Information

Figure S1 **Confounding effect of the number of SNPs per gene (Type 1 Diabetes).** Multiple test statistics are combined for each gene using three different methods (maxT, meanT, topQ). For each method, the gene-wide test statistic is correlated with the number of SNPs per gene. For these histograms, genes are binned according to their gene-wide test statistic (left axis). The red dots show the mean number of SNPs per gene for every bin (right axis).
(TIFF)

Figure S2 **Distribution of empirical p-value (** ***p_emp_*** **) for Type 1 Diabetes from 500,000 permutations of the disease labels.** Genes were assigned to 50 bins according to their *p_emp_*. Histogram shows the number of genes with *p_emp_* values (left axis). The red line shows the mean number of SNPs per gene for every bin (right axis). In contrast to the gene-wide test statistics we observe no correlation of the number of SNPs per gene with *p_emp_* for any method. We observe an increase of genes with very low *p_emp_* values caused by the actual association signal.
(TIFF)

Figure S3 **Empirical p-values vs. uncontrolled p-values (Type 1 Diabetes).** For each gene the *p_emp_* is plotted against the uncontrolled p-value (based on the gene-wide test statistic). Each point represents a gene and is coloured according to the number of SNPs assigned to a gene (*n*). Genes with few SNPs have *p_emp_* values similar to the uncontrolled p-value and therefore cluster along the diagonal. For genes with higher number of SNPs the distribution depends on the method to combine test statistics.
(TIFF)

Figure S4 **Distribution of the number of SNPs assigned to genes.** We assigned SNPs on the Affymetrix 500K genotyping array to protein-coding genes. SNPs were assigned to a gene if they are located within the transcribed region or within a 40 kilobase flanking window around the transcribed region. In addition SNPs in linkage disequilibrium (LD, r2>0.8) with these SNPs were included.
(TIFF)

Figure S5 **Effect of Linkage Disequilibrium (LD).** Gene ranks after assigning SNPs to genes based on genomic distance only are plotted against gene ranks after assigning SNPs to genes based on genomic distance and linkage disequilibrium (LD, r2>0.8). The top 500 ranks are compared for CD and T1D and all three methods to derive pemp-values.
(TIFF)

Figure S6 **Effect of Population Stratification.** Gene ranks based on an armitage trend test are plotted against gene ranks based on logistic regression and adjusting for two principal components. The top 500 ranks are compared for CD and T1D and all three methods to derive pemp-values.
(TIFF)

Table S1 **Pairwise Spearman rank correlation for the different methods to combine test statistics before and after controlling for multiple hypothesis testing for Crohn's Disease.** For the correlation the top 500 genes were considered.
(DOC)

Table S2 **Pairwise Spearman rank correlation for the different methods to combine test statistics before and after controlling for multiple hypothesis testing for Type 1 Diabetes.** For the correlation the top 500 genes were considered.
(DOC)

Table S3 **Replicated Disease Genes for Type 1 Diabetes (T1D) and their ranks for each method.**
(DOC)

Table S4 **The top 30 genes for Type 1 Diabetes (T1D) ranked using the maxT method.** Genes are ordered by chromosome and genomic position; n denominates the number of SNPs per gene. The last three columns show the corresponding ranks for the three methods. *italics:* genes that are within the true positive list.
(DOC)
