# Supplementary figures and images for: From SNPs to Genes: Disease Association at the Gene Level
Source: PLoS One. 2011 Jun 30;6(6):e20133. doi: 10.1371/journal.pone.0020133 (PMC3128073; doi:10.1371/journal.pone.0020133)

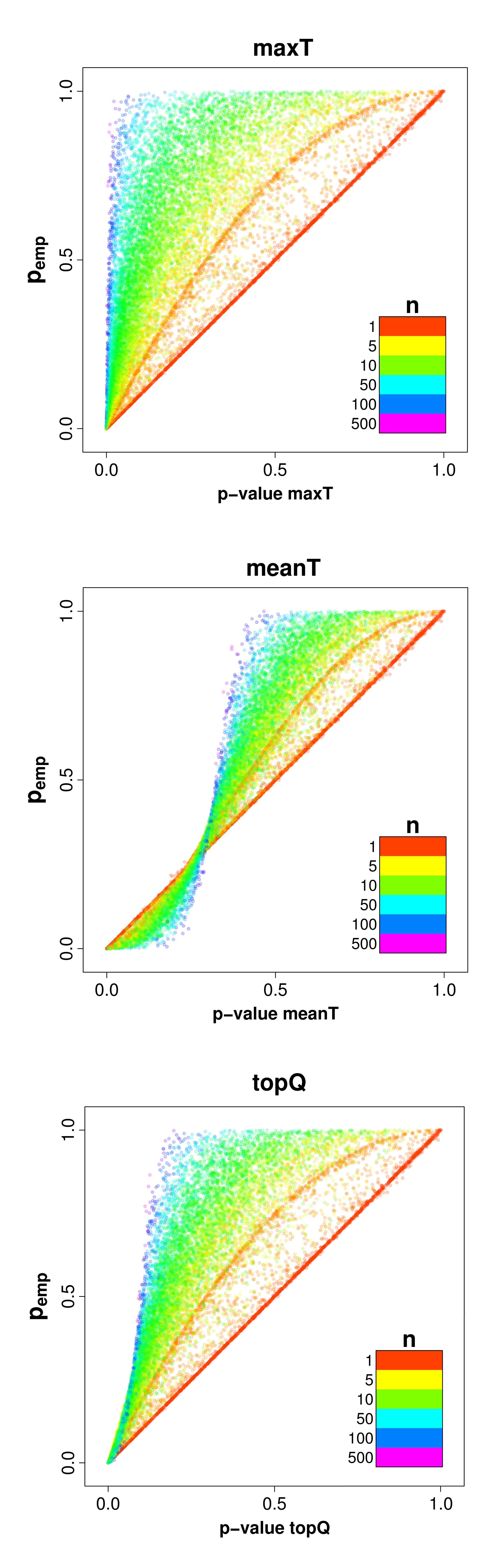

Supplement: Figure S3 — Empirical p-values vs. uncontrolled p-values (Type 1 Diabetes). For each gene the pemp is plotted against the uncontrolled p-value (based on the gene-wide test statistic). Each point represents a gene and is coloured according to the number of SNPs assigned to a gene (n). Genes with few SNPs have pemp values similar to the uncontrolled p-value and therefore cluster along the diagonal. For genes with higher number of SNPs the distribution depends on the method to combine test statistics. (TIFF) [file pone.0020133.s003.tiff]
