# Supplementary material for: From SNPs to Genes: Disease Association at the Gene Level
Source: PLoS One. 2011 Jun 30;6(6):e20133. doi: 10.1371/journal.pone.0020133 (PMC3128073; doi:10.1371/journal.pone.0020133)
Supplement: Table S1 — Pairwise Spearman rank correlation for the different methods to combine test statistics before and after controlling for multiple hypothesis testing for Crohn's Disease. For the correlation the top 500 genes were considered. (DOC) [file pone.0020133.s007.doc]

**Table S1**

| **CD** |  | **uncontrolled** | | | **controlled** | | |
| --- | --- | --- | --- | --- | --- | --- | --- |
|  |  | maxT | meanT | topQ | maxT | meanT | topQ |
| maxT | uncontrolled | 1.00 | 0.08 | 0.74 | 0.73 | 0.52 | 0.71 |
| meanT | uncontrolled | 0.08 | 1.00 | 0.49 | 0.55 | 0.73 | 0.58 |
| topQ | uncontrolled | 0.74 | 0.49 | 1.00 | 0.78 | 0.73 | 0.90 |
| maxT | controlled | 0.73 | 0.55 | 0.78 | 1.00 | 0.60 | 0.83 |
| meanT | controlled | 0.52 | 0.73 | 0.73 | 0.60 | 1.00 | 0.78 |
| topQ | controlled | 0.71 | 0.58 | 0.90 | 0.83 | 0.78 | 1.00 |
