# Supplementary material for: From SNPs to Genes: Disease Association at the Gene Level
Source: PLoS One. 2011 Jun 30;6(6):e20133. doi: 10.1371/journal.pone.0020133 (PMC3128073; doi:10.1371/journal.pone.0020133)
Supplement: Table S2 — Pairwise Spearman rank correlation for the different methods to combine test statistics before and after controlling for multiple hypothesis testing for Type 1 Diabetes. For the correlation the top 500 genes were considered. (DOC) [file pone.0020133.s008.doc]

**Table S2**

| **T1D** |  | **uncontrolled** | | | **controlled** | | |
| --- | --- | --- | --- | --- | --- | --- | --- |
|  |  | maxT | meanT | topQ | maxT | meanT | topQ |
| maxT | uncontrolled | 1.00 | 0.08 | 0.73 | 0.76 | 0.50 | 0.73 |
| meanT | uncontrolled | 0.08 | 1.00 | 0.49 | 0.50 | 0.74 | 0.54 |
| topQ | uncontrolled | 0.73 | 0.49 | 1.00 | 0.77 | 0.69 | 0.91 |
| maxT | controlled | 0.76 | 0.50 | 0.77 | 1.00 | 0.57 | 0.82 |
| meanT | controlled | 0.50 | 0.74 | 0.69 | 0.57 | 1.00 | 0.75 |
| topQ | controlled | 0.73 | 0.54 | 0.91 | 0.82 | 0.75 | 1.00 |
