# Supplementary material for: From SNPs to Genes: Disease Association at the Gene Level
Source: PLoS One. 2011 Jun 30;6(6):e20133. doi: 10.1371/journal.pone.0020133 (PMC3128073; doi:10.1371/journal.pone.0020133)
Supplement: Table S3 — Replicated Disease Genes for Type 1 Diabetes (T1D) and their ranks for each method. (DOC) [file pone.0020133.s009.doc]

Table S3

| **HGNC symbol** | **Number of SNPs per gene n** | **rank maxT** | **rank meanT** | **Rank topQ** |
| --- | --- | --- | --- | --- |
|  |  |  |  |  |
| ***PTPN22*** | 4 | 3 | 11 | 3 |
| ***ERBB3*** | 2 | 4 | 2 | 4 |
| ***CLEC16A*** | 45 | 20 | 19 | 20 |
| ***SH2B3*** | 2 | 26 | 33 | 26 |
| ***PTPN2*** | 20 | 27 | 92 | 42 |
| ***IL2RA*** | 20 | 36 | 71 | 41 |
| ***CTLA4*** | 15 | 44 | 29 | 31 |
| ***PGM1*** | 29 | 45 | 36 | 35 |
| ***IL2*** | 4 | 76 | 81 | 82 |
| ***C1QTNF6*** | 7 | 160 | 445 | 111 |
| ***CTSH*** | 6 | 218 | 441 | 411 |
| ***CD69*** | 14 | 249 | 78 | 58 |
| ***IFIH1*** | 6 | 269 | 1,088 | 223 |
| ***UBASH3A*** | 12 | 445 | 416 | 251 |
| ***C6orf173*** | 3 | 504 | 620 | 494 |
| ***IL7R*** | 15 | 552 | 1,718 | 293 |
| ***IL10*** | 3 | 894 | 133 | 876 |
| ***RGS1*** | 15 | 1,854 | 967 | 1,206 |
| ***CCR5*** | 6 | 1,872 | 862 | 1,503 |
| ***BACH2*** | 49 | 2,788 | 501 | 554 |
| ***GLIS3*** | 169 | 3,923 | 10,816 | 10,249 |
| ***IL18RAP*** | 15 | 5,962 | 9,082 | 7,442 |
| ***TNFAIP3*** | 10 | 6,289 | 6,122 | 6,058 |
| ***COBL*** | 46 | 6,681 | 1,961 | 2,181 |
| ***PRKCQ*** | 53 | 7,013 | 6,567 | 6,148 |
| ***CD226*** | 14 | 12,588 | 11,503 | 12,206 |
| ***ORMDL3*** | 1 | 14,370 | 14,328 | 14,333 |
