# Supplementary material for: From SNPs to Genes: Disease Association at the Gene Level
Source: PLoS One. 2011 Jun 30;6(6):e20133. doi: 10.1371/journal.pone.0020133 (PMC3128073; doi:10.1371/journal.pone.0020133)
Supplement: Table S4 — The top 30 genes for Type 1 Diabetes (T1D) ranked using the maxT method. Genes are ordered by chromosome and genomic position; n denominates the number of SNPs per gene. The last three columns show the corresponding ranks for the three methods. italics: genes that are within the true positive list. (DOC) [file pone.0020133.s010.doc]

Table S4

| **HGNC symbol** | **Chr location** | **Region (Mb)** | **n** | **p-value maxT** | **p-value meanT** | **p-value topQ** | **rank maxT** | **rank meanT** | **rank topQ** |
| --- | --- | --- | --- | --- | --- | --- | --- | --- | --- |
| MAGI3 | 1p12 | 113.93-114.23 | 32 | >2.0E-06 | >2.0E-06 | >2.0E-06 | 6 | 12 | 6 |
| PHTF1 | 1p13 | 114.24-114.30 | 1 | >2.0E-06 | >2.0E-06 | >2.0E-06 | 5 | 3 | 5 |
| RSBN1 | 1p13 | 114.30-114.36 | 4 | >2.0E-06 | >2.0E-06 | >2.0E-06 | 1 | 1 | 1 |
| ***PTPN22*** | 1p13 | 114.36-114.41 | 4 | >2.0E-06 | >2.0E-06 | >2.0E-06 | 3 | 11 | 3 |
| AP4B1 | 1p13 | 114.44-114.45 | 3 | 2.0E-06 | 2.0E-06 | 2.0E-06 | 12 | 15 | 12 |
| HIPK1 | 1p13 | 114.47-114.52 | 3 | 4.0E-06 | 8.5E-05 | 4.0E-06 | 14 | 30 | 17 |
| PIK3C2B | 1q32 | 204.39-204.46 | 9 | 1.1E-04 | 7.6E-02 | 1.9E-02 | 30 | 1,420 | 459 |
| MOGS | 2p13 | 74.69-74.69 | 1 | 1.1E-04 | 1.1E-04 | 1.1E-04 | 30 | 32 | 33 |
| DQX1 | 2p13 | 74.75-74.75 | 1 | 4.0E-05 | 4.0E-05 | 4.0E-05 | 25 | 27 | 24 |
| ADAD1 | 4q27 | 123.30-123.35 | 4 | 2.0E-06 | >2.0E-06 | 2.0E-06 | 11 | 13 | 11 |
| HIST1H2BA | 6p22 | 25.73-25.73 | 3 | 6.3E-05 | 4.9E-03 | 6.3E-05 | 28 | 169 | 28 |
| SLC17A3 | 6p21 | 25.85-25.87 | 18 | 4.0E-06 | 4.0E-06 | 2.0E-06 | 16 | 17 | 15 |
| LOC100131294 | 12q13 | 56.37-56.39 | 4 | >2.0E-06 | >2.0E-06 | >2.0E-06 | 7 | 10 | 7 |
| ***ERBB3*** | 12q13 | 56.47-56.50 | 2 | >2.0E-06 | >2.0E-06 | >2.0E-06 | 4 | 2 | 4 |
| MYL2 | 12q24 | 111.35-111.36 | 3 | 1.2E-05 | 9.9E-06 | 1.2E-05 | 22 | 21 | 22 |
| LOC100131138 | 12q24 | 111.38-111.38 | 11 | 4.0E-06 | 4.0E-06 | 4.0E-06 | 17 | 16 | 18 |
| CUX2 | 12q24 | 111.47-111.79 | 18 | 4.0E-06 | 4.0E-06 | 2.0E-06 | 15 | 18 | 16 |
| ***SH2B3*** | 12q24 | 111.84-111.89 | 2 | 5.6E-05 | 1.2E-04 | 5.6E-05 | 26 | 33 | 26 |
| ATXN2 | 12q24 | 111.89-112.04 | 2 | 7.7E-05 | 5.1E-04 | 7.7E-05 | 29 | 54 | 30 |
| BRAP | 12q24 | 112.08-112.12 | 2 | 2.0E-06 | >2.0E-06 | 2.0E-06 | 13 | 7 | 14 |
| ACAD10 | 12q24 | 112.12-112.19 | 8 | >2.0E-06 | >2.0E-06 | >2.0E-06 | 8 | 8 | 10 |
| ALDH2 | 12q24 | 112.20-112.25 | 2 | >2.0E-06 | >2.0E-06 | >2.0E-06 | 9 | 6 | 8 |
| MAPKAPK5 | 12q24 | 112.28-112.33 | 7 | 4.0E-06 | >2.0E-06 | 2.0E-06 | 19 | 9 | 13 |
| TMEM116 | 12q24 | 112.37-112.45 | 6 | 1.8E-05 | 2.0E-06 | 4.0E-06 | 24 | 14 | 19 |
| C12orf30 | 12q24 | 112.46-112.55 | 3 | >2.0E-06 | >2.0E-06 | >2.0E-06 | 2 | 4 | 2 |
| TRAFD1 | 12q24 | 112.56-112.59 | 2 | 6.0E-06 | 2.2E-05 | 6.0E-06 | 21 | 23 | 21 |
| C12orf51 | 12q24 | 112.60-112.74 | 5 | 4.0E-06 | 2.2E-05 | 3.6E-05 | 18 | 22 | 23 |
| PTPN11 | 12q24 | 112.86-112.95 | 6 | 1.6E-05 | 6.5E-05 | 6.5E-05 | 23 | 28 | 29 |
| ***CLEC16A*** | 16p13 | 11.04-11.28 | 45 | 6.0E-06 | 6.0E-06 | 4.0E-06 | 20 | 19 | 20 |
| ***PTPN2*** | 18p11 | 12.79-12.88 | 20 | 6.3E-05 | 1.7E-03 | 2.7E-04 | 27 | 92 | 42 |
